# Self-reported Health Problems of Professional Dancers from Five German Opera Houses or State Theatres: A Prospective Study with Weekly Follow-ups during One Season

**DOI:** 10.1186/s40798-024-00782-w

**Published:** 2024-11-09

**Authors:** Astrid Junge, Rogier M van Rijn, Janine H Stubbe, Anja Hauschild

**Affiliations:** 1 Center for Health in Performing Arts, MSH Medical School Hamburg, Hamburg, Germany; 2 Institute of Interdisciplinary Exercise Science and Sports Medicine, MSH Medical School Hamburg, Am Kaiserkai 1, 20457 Hamburg, Germany; 3 Codarts Rotterdam, University of the Arts, Rotterdam, The Netherlands; 4 Performing artist and Athlete Research Lab (PEARL), Rotterdam, The Netherlands; 5 Center for Rehabilitation and Sports Medicine, BG Klinikum Hamburg, Hamburg, Germany

**Keywords:** Musculoskeletal pain, Injury, Complaints, Performing artists, Workload

## Abstract

**Background:**

Most studies on injuries of professional dancers used a medical-attention and/or time-loss definition and did not analyse all health problems. Further, almost all studies included just one company. The aim was to analyse all self-reported health problems of professional ballet and contemporary dancers during one season and compare sexes and five companies in Germany.

**Methods:**

Dancers of five professional companies completed weekly health questionnaires during the season (September 2022 to June 2023). Numerical rating scales were used for severity of all health problems, musculoskeletal pain, impairment of the ability to dance at full potential, physical and mental workload in the previous seven days. If the severity of all health problems were rated greater than “0”, the dancers were asked to report the type and consequences of their most severe health problem.

**Results:**

During 43 weeks, 98 dancers (39.8% male) completed 3123 weekly reports (response rate 74.1%). The season prevalence of any health problem was 100% and of time-loss health problems 74.5%. The average weekly prevalence of any health problem was 62.7%, of musculoskeletal pain 83.4% and of impaired ability to dance at full potential, due to health problem 48.6%. While the season prevalence and type of health problems was similar between sexes, the average weekly prevalence of severe health problems was higher in female than in male dancers (Chi^2^ = 23.2; *p* < .001), and female dancers saw a qualified health professional more often than male dancers (Chi^2^ = 19.5; *p* < .001). Companies differed in almost all investigated variables, with more health problems in companies where more dancers rated their workload higher than “ideal”.

**Conclusion:**

Health problems are frequent in professional dancers and affect their ability to dance. Future studies should analyse the impact of physical and mental workload on health problems.

**Supplementary Information:**

The online version contains supplementary material available at 10.1186/s40798-024-00782-w.

## Key Points

• Professional dancers reported a health problem more often than in every second week (63%). Almost two thirds of the dancers (*n* = 63, 64.3%) reported a severe health problem and three quarters (*n* = 73, 74.5%) felt unable to dance due to a health problem at least one day during the season.

• The season prevalence of health problems was similar in female and male dancers, however the average weekly prevalence of severe health problems was higher in female than in male dancers, and female dancers saw a qualified health professional more often than male dancers.

• Companies differed significantly in the average weekly prevalence of health problems and the average severity rating of health problems during the season. Health problems were more frequent and more severe in companies where more dancers rated their workload higher than “ideal”.

**Supplementary Information:**

The online version contains supplementary material available at 10.1186/s40798-024-00782-w.

## Background

Several studies have reported the incidence, prevalence and characteristics of injuries of professional dancers [1–6], however almost all used a medical-attention and/or time-loss definition. Several parameters influence whether or not an injury receives medical attention or results in time-loss by, e.g. the availability of in-house medical staff, access to the public health care system, timely appointments, pressure to perform. In an international retrospective survey on 260 professional dancers more than 15% of all injured dancers stated that they had not reported their injury for various reasons [3]. Thus, time-loss and medical-attention injury definitions underestimate the injury burden [7–10]. For example, in a study on 452 pre-professional ballet dancers (mean age 15 years) the prevalence of time-loss injuries was 32.1% but the prevalence of all-complaint injuries was more than twice as high (67.4%).^7^ Therefore, an all-complaints injury definition seems more adequate to assess the full burden of health problems.

Self-reports of health problems [11–14] can not only be used to get a more comprehensive picture of the burden of all complaints, it can also be implemented when no in-house medical staff is available for documentation. Such self-reports have been implemented in a few studies on pre-professional dancers [7, 14–17], but just one study on self-reported health problems of professional dancers was found in the literature [18]. 

A challenge of self-reports on health problems by professional dancers is the distinction between injury and pain. In a qualitative study on perception of injuries of professional dancers “participants defined an injury based mainly on dance performance limitations, while pain and time loss reflected injury severity” [19]. This observation is in agreement with another qualitative study reporting that professional dancers had difficulties in classifying pain as an injury when they were still able to perform [13]. It is, therefore, plausible to ask dancers separate questions on the presence or intensity of pain, other health problems and limitations of the ability to perform [18]. 

In addition, most studies on health problems of professional dancers included just one company. Comparisons between studies are difficult due to differences in the applied methodology (e.g. injury definition, method of data collection, study period). A cross-sectional study on professional ballet and contemporary dancers that reported the injury prevalences of different companies showed large differences, e.g. the prevalence of persisting injuries ranged from 9.1–35.7%.^3^

The primary aim, therefore, was to analyse the prevalence and severity of self-reported health problems, musculoskeletal pain, and their effect on dance ability of professional dancers from five German companies during the season. Secondary aims were to compare these variables between female and male dancers and between the companies.

## Methods

All dancers of five companies of German opera houses or states theatres (*n* = 219) were asked to participate in a comprehensive health and performance screening at the start of the season 2022/23 and then report their health problems weekly using the Performing artist and Athlete Health Monitor (PAHM [13, 14, 18]) during 43 weeks of the season (September 2022 to June 2023). Two companies had less than twenty dancers (“small”), one company between twenty and fifty (“medium”), and two more than fifty dancers (“large”). Four companies danced primarily ballet, and one company contemporary.

The PAHM is a web-based system to record health problems (i.e. injuries, illnesses and mental health problems). The PAHM was pseudonymous (i.e. dancers used a personal code). Only the individual dancer and two authors of the study (AH, RMvR) knew the match of code and name and kept this information strictly confidential and in accordance with the German data protection laws. The health record started with numerical rating scales (NRS) on physical and mental workload (ranging from “much too low” (-5) over “ideal” (0) to much too high” (+ 5)), severity of all health problems, musculoskeletal pain (both ranging from “not at all” (0) to “worst imaginable” (10)) and on impairment of the ability to dance at full potential due to health problems (ranging from “dance at full potential” (0) to “unable to dance” (10)) in the previous seven days. These variables were categorized based on the severity as no (NRS = 0), mild (NRS = 1–3), moderate (NRS = 4–6) or severe (NRS = 7–10). “All health problems” were defined as all kinds of pain, complaints, injuries, illnesses, and mental health problems. A health problem was defined as “time-loss” if the dancer were at least one day unable to dance (see question (d) below). Dancers who rated the severity of “all health problems” greater than “0” on the NRS were asked (a) if their most severe health problem was an injury (defined as “musculoskeletal pain, complaints or injury, e.g. sore muscles, ankle sprain, concussion”), illness (defined as “illness or physical symptoms, e.g. influenza, diarrhoea, headache, menstrual pain”) and/or mental health problem (defined as “mental health issue, e.g. performance anxiety, depression”), (b) if they saw a physician, physiotherapist, psychologist or another qualified medical practitioner because of their health problem(s), (c) on how many days the health problem(s) had affected their ability to dance at full potential and (d) on how many days they were completely unable to train, rehearse or perform due to their health problem(s) related to the last seven days. Dancers were asked every Friday to fill in the PAHM, if they did not respond within two days, they received a reminder.

All dancers were informed about the content and aims of the study, and those participating gave written informed consent before the start of the study. The study received ethical approval (MSH 2021/137) of the MSH Medical School Hamburg, Germany. The study was conducted in accordance with the Declaration of Helsinki.

The dancers were included in the analysis if they filled in at least 25% of the weekly health reports [11, 20, 21]. All data were processed using Excel (version 16.74, Microsoft, Redmond, U.S.A.) and SPSS (version 27, IBM Armonk, U.S.A.). Data were analysed on level of dancers (e.g. season prevalence) and on the level of weekly reports (e.g. average severity ratings). Missing data were excluded from the analyses, e.g. all percentage were based on the valid numbers. Results were reported as number with percentages or mean with standard deviation. Season prevalence was calculated by dividing the number of dancers who reported the respective variables (e.g. musculoskeletal pain, any health problem) at least once during the season by the number of all dancers and expressed as percentage. Average weekly prevalence was calculated by dividing the number of weekly reports with the respective variable by the number of all weekly reports received and expressed as percentage. Statistical methods applied were frequencies, means, Pearson correlation for analysis of association between the severity of health problems, musculoskeletal pain and ability to dance, Shapiro-Wilk-test for normality distribution, chi^2^-test for comparison of nominal scaled variables, Mann-Whitney-U-test of interval scaled variables for comparison between sexes, and Kruskal-Wallis-test for comparison of interval scaled variables between companies. Correlations were defined as low (*r* < .50), moderate (*r* = .50 - .70) or high (*r* > .70). Significance was accepted at *p* < .05. The level of significance for the comparison of female and male dancers and for the comparison of the companies was p *≤* .002 according to Bonferroni correction for multiple testing.

## Results

### Study Group and Response Rate

Of the 120 professional dancers from five professional dance companies who agreed to participate in the study, 98 dancers (81.7%) filled in at least 25% of the weekly health reports. Fifty-nine (60.2%) dancers were females and 39 (39.8%) males. The dancers were on average 26.8 years old (sd = 5.9, range 18–42 years) without difference between sexes or companies. All dancers had a professional dance education and were full time employed (40 h/week) at their company, 25 (25.5%) in small, 26 (26.5%) in medium, and 47 (48.0%) in large companies. The dancers usually worked six days per week, and the companies had between 54 and 79 performances (incl. 3 to 6 premieres) during the study period.

The 98 dancers filled in a total of 3123 weekly reports during the 43 weeks of the season, covering a total of 21,861 dancer-days. On average, every dancer returned 31.9 reports which is a response rate of 74.1%. The response rate was higher in male than in female dancers (Table 1) and varied between companies between 65.9% and 90.5% (Table 2).

**Table 1:** Comparison of the weekly reports from female and male professional dancers regarding the prevalence and severity of health problems, type of most severe health problem, medical attention, days with impaired dancing ability, and physical and mental workload in the previous seven days during 43 weeks of the season

	All (*n* = 98)	Female (*n* = 59)	Male (*n* = 39)	Comparison	
**Response rate**	**N (%)**	**N (%)**	**N (%)**	**Chi** ^**2**^	**p-value**
Weekly health reports returned	3123 (74.1%)	1830 (72.1%)	1293 (77.1%)	13.0	**< 0.001**
Dancer-days covered	21,861	12,810	9051		
**Season prevalence**	**%**	**%**	**%**	**Chi** ^**2**^	**p-value**
Any health problem^†,§^	100%	100%	100%	no difference	
Any musculoskeletal pain^†,§^	100%	100%	100%	no difference	
Any impaired ability to dance^‡,§^	90.7%	88.1%	94.7%	1.28	0.26
Severe health problem^†,¶^	64.3%	67.8%	59.0%	0.80	0.37
Severe musculoskeletal pain^†,¶^	76.5%	79.7%	71.8%	0.81	0.37
Severely impaired ability to dance^§,¶^	60.8%	62.7%	57.9%	2.19	0.14
Time-loss health problem	74.5%	72.9%	76.9%	0.20	0.65
**Average weekly prevalence**	**%**	**%**	**%**	**Chi** ^**2**^	**p-value**
Any health problem^†,§^	62.7%	62.0%	63.8%	1.01	0.32
Any musculoskeletal pain^†,§^	83.4%	84.9%	81.1%	7.81	0.003
Any impaired ability to dance^‡,§^	48.6%	48.2%	49.2%	0.24	0.62
Severe health problem^†,¶^	6.8%	8.6%	4.2%	23.2	**< 0.001**
Severe musculoskeletal pain^†,¶^	10.6%	10.9%	10.6%	0.26	0.61
Severely impaired ability to dance^§,¶^	8.8%	10.3%	6.7%	9.15	**0.002**
Time-loss health problem	10.2%	11.0%	9.0%	3.10	0.08
**Average severity rating of**	**Mean (sd)**	**Mean (sd)**	**Mean (sd)**	**U-test**	**p-value**
All health problems^§^	2.15 (2.33)	2.22 (2.46)	2.05 (2.13)	3.88	0.05
Musculoskeletal pain^§^	3.02 (2.33)	3.00 (2.32)	3.03 (2.35)	0.15	0.70
Impaired ability to dance^¶^	1.87 (2.70)	1.89 (2.81)	1.83 (2.53)	2.28	0.60
**Average days per week dancers were …**	**Mean (sd)**	**Mean (sd)**	**Mean (sd)**	**U-test**	**p-value**
Impaired in their ability to dance	1.59 (2.50)	1.56 (2.45)	1.63 (2.58)	0.65	0.42
Unable to dance	0.38 (1.39)	0.44 (1.50)	0.30 (1.21)	7.73	**0.005**
**Type of most severe health problem** ^#^	**N (%)**	**N (%)**	**N (%)**	**Chi** ^**2**^	**p-value**
Injury	881 (76.7%)	477 (75.6%)	404 (79.4%)	4.21	0.12
Illness	151 (13.2%)	94 (14.7%)	57 (11.2%)		
Mental health problem	116 (10.1%)	68 (10.6%)	48 (9.4%)		
**Medical attention**	**N (%)**	**N (%)**	**N (%)**		
In weeks with a health problem	910 (46.7%)	574 (51.0%)	336 (40.9%)	19.5	**< 0.001**
**Physical Workload** ^*^	**N (%)**	**N (%)**	**N (%)**	**Chi** ^**2**^	**p-value**
Lower than “ideal”	610 (19.7%)	356 (19.6%)	254 (19.8%)	16.7	**< 0.001**
“ideal”	927 (29.9%)	495 (27.3%)	432 (33.7%)		
Higher than “ideal”	1560 (50.4%)	963 (53.1%)	597 (46.5%)		
**Mental Workload** ^*^	**N (%)**	**N (%)**	**N (%)**	**Chi** ^**2**^	**p-value**
Lower than “ideal”	444 (14.3%)	253 (13.9%)	191 (14.9%)	26.0	**< 0.001**
“ideal”	982 (31.7%)	516 (28.4%)	466 (36.3%)		
Higher than “ideal”	1671 (54.0%)	1045 (57.6%)	626 (48.8%)		

NRS = numerical rating scale; ^†^ NRS ranging from “dance at full potential” (0) to “unable to dance” (10); ^‡^ NRS ranging from “not at all” (0) to “worst imaginable” (10); ^§^ NRS > 0; ^¶^ NRS = 7–10; ^#^ information is not available for all weeks, ^*^ NRS ranging from “much too low” (-5) over “ideal” (0) to much too high” (+ 5). Results significant at p ≤ .002 (Bonferroni corrected) are highlighted in bold

**Table 2:** Comparison of the weekly reports from professional dancers of five companies with respect to average weekly prevalence of health problems, days with impairment due to a health problem, the dancers´ average weekly rating of the severity of health problems as well as of physical and mental workload in the previous seven days during 43 weeks of the season. Highest values in **bold**, lowest values in *italics*

Company	A	B	C	D	E	Comparison	
**Sample characteristics**	**%**	**%**	**%**	**%**	**%**	**Chi** ^**2**^	**p-value**
% of female dancers in the study^†^	*50.0%*	61.5%	**67.7%**	61.5%	58.6%	16.8	0.002
% of returned weekly health reports^†^	76.7%	**90.5%**	68.3%	*65.9%*	76.6%		
**Season prevalence**	**%**	**%**	**%**	**%**	**%**	**Chi** ^**2**^	**p-value**
Any health problem^‡,¶^	100%	100%	100%	100%	100%	no difference	
Any musculoskeletal pain^‡,¶^	100%	100%	100%	100%	100%	no difference	
Any impaired ability to dance^§,¶^	**100%**	*84.6%*	88.9%	92.3%	89.3%	2.01	0.73
Severe health problem^‡,#^	75.9%	61.5%	**83.3%**	61.5%	*51.7%*	4.71	0.32
Severe musculoskeletal pain^‡,#^	83.3%	**92.3%**	83.3%	73.1%	*65.5%*	5.57	0.23
Severely impaired ability to dance^§,#^	**91.7%**	69.2%	61.1%	53.8%	*50.0%*	6.73	0.15
Time-loss health problem	**100%**	84.6%	*61.1%*	73.1%	69.0%	7.00	0.14
7 or more days unable to dance	41.7%	**46.2%**	*27.8%*	34.6%	34.5%	1.33	0.86
**Average weekly prevalence**	**%**	**%**	**%**	**%**	**%**	**Chi** ^**2**^	**p-value**
Any health problem^‡,¶^	**84.3%**	72.8%	*48.6%*	56.2%	61.3%	159.4	< 0.001
Any musculoskeletal pain^‡,¶^	**95.2%**	90.3%	*72.3%*	82.6%	81.5%	105.9	< 0.001
Any impaired ability to dance^§,¶^	**67.7%**	53.3%	*42.3%*	45.1%	44.0%	59.2	< 0.001
Severe health problem^‡,#^	**10.4%**	9.3%	**10.4%**	4.7%	*3.5%*	46.3	< 0.001
Severe musculoskeletal pain^‡,#^	12.7%	**17.7%**	13.3%	9.6%	*5.5%*	59.4	< 0.001
Severely impaired ability to dance^§,#^	**19.8%**	8.6%	11.8%	5.3%	*5.2%*	66.0	< 0.001
Time-loss health problem	**17.7%**	10.7%	7.9%	11.3%	*7.3%*	36.7	< 0.001
7 or more days unable to dance	**6.6%**	3.4%	2.8%	2.6%	*2.3%*	18.3	0.001
**Average severity rating of**	**Mean (sd)**	**Mean (sd)**	**Mean (sd)**	**Mean (sd)**	**Mean (sd)**	**KWT**	**p-value**
All health problems^¶^	**2.98 (2.29)**	2.67 (2.49)	1.97 (2.61)	*1.88 (2.19)*	*1.85 (2.06)*	129.7	< 0.001
Musculoskeletal pain^¶^	3.37 (2.15)	**3.77 (2.55)**	2.79 (2.60)	3.12 (2.27)	*2.52 (2.02)*	105.8	< 0.001
Impaired ability to dance^#^	**3.17 (3.29)**	2.10 (2.73)	1.88 (2.88)	1.54 (2.39)	*1.43 (2.32)*	85.2	< 0.001
**Average days per week dancers were …**	**Mean (sd)**	**Mean (sd)**	**Mean (sd)**	**Mean (sd)**	**Mean (sd)**	**KWT**	**p-value**
Impaired in their ability to dance	**2.55 (2.95)**	1.91 (2.44)	1.81 (2.75)	1.35 (2.28)	*1.08 (2.19)*	121.6	< 0.001
Unable to dance	**0.67 (1.83)**	0.41 (1.44)	0.32 (1.31)	0.39 (1.35)	*0.28 (1.21)*	18.4	0.001
**Physical Workload** ^†,*^	**%**	**%**	**%**	**%**	**%**	**Chi** ^**2**^	**p-value**
Lower than “ideal” (NRS − 5 to -1)	*15.7%*	17.5%	*15.6%*	19.1%	**25.2%**	51.2	< 0.001
“ideal” (NRS = 0)	**23.9%**	27.4%	31.7%	*33.6%*	30.0%		
Higher than “ideal” (NRS + 1 to + 5)	**60.4%**	55.1%	52.7%	47.2%	*44.8%*		
**Mental Workload** ^†,*^	**%**	**%**	**%**	**%**	**%**	**Chi** ^**2**^	**p-value**
Lower than “ideal” (NRS − 5 to -1)	12.9%	15.5%	*9.7%*	10.2%	**20.0%**	60.7	< 0.001
“ideal” (NRS = 0)	**26.1%**	27.6%	*36.3%*	34.8%	31.2%		
Higher than “ideal” (NRS + 1 to + 5)	**60.9%**	56.9%	54.0%	55.0%	*48.7%*		

NRS = numerical rating scale; ^†^ For confidentiality reasons only percentages are presented; ‡ NRS ranging from “not at all” (0) to “worst imaginable” (10); § NRS ranging from “dance at full potential” (0) to “unable to dance” (10); ¶ NRS > 0; # NRS = 7–10; * NRS ranging from “much too low” (-5) over “ideal” (0) to much too high” (+ 5); KWT = Kruskal-Wallis-Test. All significant results remained significant after Bonferroni correction at p *≤* .002)

### Health Problems and Impaired Ability to Dance

The season prevalence was 100% for all health problems and 74.5% for time-loss health problems. More than a third of the dancers (35.7%) had health problems that lasted one week or longer. All dancers reported some degree of musculoskeletal pain, 90.0% (*n* = 88) an impairment of their ability to dance at full potential due to their health problem, and almost two thirds of the dancers (*n* = 63, 64.3%) a severe health problem during the season (Table 1).

**Fig. 1:**
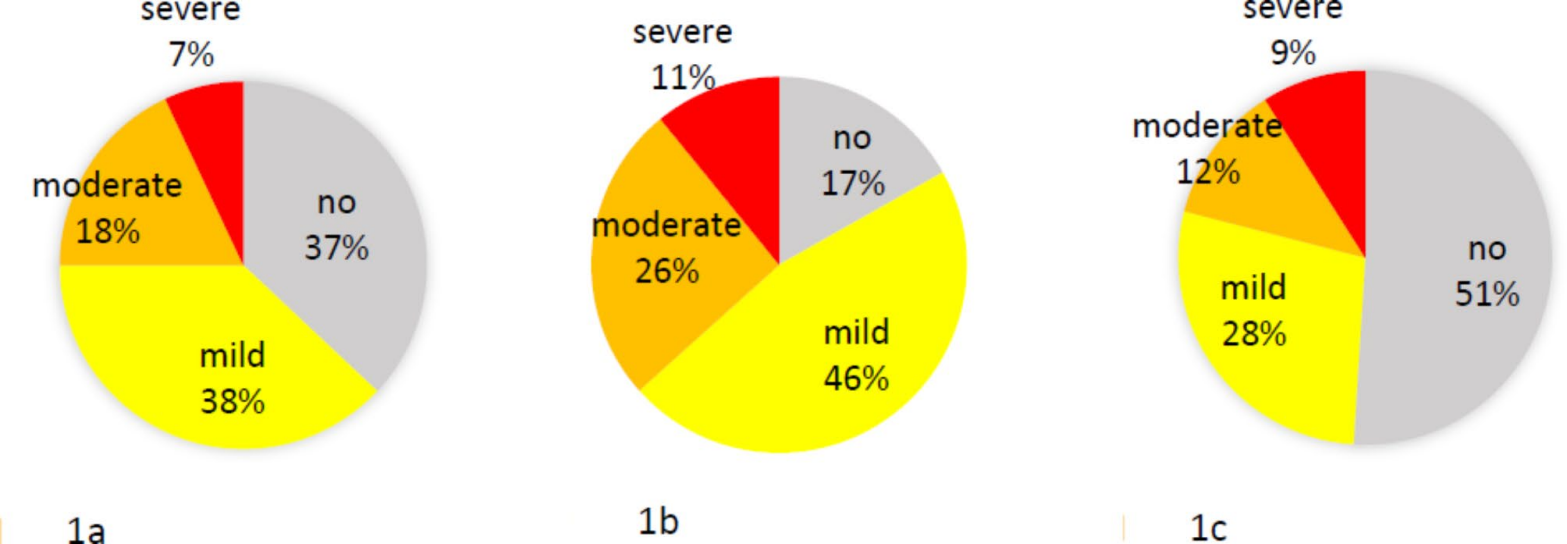
**a**, **b**, **c**: Average weekly prevalence of different severity of (**a**) all health problems, (**b**) musculoskeletal pain and (**c**) impaired ability to dance at full potential due to all health problems in the last 7 days on numerical rating scales (NRS) ranging from 0 to 10 (no: NRS = 0; mild: NRS = 1 to 3; moderate: NRS = 4 to 6; severe: NRS = 7 to 10)

The average weekly prevalence of any health problem was 62.7% and of time-loss health problems 10.2%. The dancers reported some degree of musculoskeletal pain in 83.4% and severe musculoskeletal pain in 10.6% of the weeks. In almost half of the reports (48.6%) the dancers felt affected in their ability to dance at full potential due to health problems. For details on the severity of all health problems, musculoskeletal pain and impaired ability to dance see Fig. 1; Table 1. The severity of musculoskeletal pain correlated moderately with the severity of all health problems (*r* = .57, *p* < .01) and less with the rating of impaired ability to dance at full potential (*r* = .42, *p* < .01), while the correlation of the latter two variables was high (*r* = .77, *p* < .01).

The most severe health problems were classified as injuries in 881 (76.7%) reports, as illnesses in 151 (13.2%) and as mental health problems in 116 (10.1%) reports (this information was not available for all weeks). In 46.7% of the weeks when the dancers reported a health problem, they saw a qualified health professional: physiotherapist (751, 38.6%), physician (194, 10.0%) or another health professional (60, 3.1%), mainly a psychotherapist, or an osteopath (multiple answers possible). The health problems resulted in 4955 days when a dancer was not able to dance at full potential, including 1114 days when a dancer was completely unable to dance, this is equivalent to 22.7% respectively 5.5% of the 21,861 days documented in the weekly reports.

### Differences between Female and Male Dancers

Female and male dancers were similar in almost all variables investigated (Table 1). The average weekly prevalence of severe health problems was about twice as high in female than in male dancers (Chi^2^ = 23.2; *p* < .001) and female dancers saw a qualified health professional in more weeks than male dancers (Chi^2^ = 19.5; *p* < .001). Further, more female than male dancers rated their physical (Chi^2^ = 16.7; *p* < .001) and mental workload (Chi^2^ = 26.0; *p* < .001) higher than “ideal” and fewer female than male dancers as “ideal”.

### Differences between Companies

The response rate and the proportion of participating female dancers varied between companies (Table 2). However, no related systematic pattern was observed, i.e. the response rate did not seem to influence the differences between companies in health problems and workload. The season prevalence of severe musculoskeletal pain (range: 65.5–92.3%) and severe health problems (range: 51.7–83.3%) differed between companies in a clinically signficant, but not statistically significant way. Significant differences between companies were found for the average weekly prevalence and all other investigated variables. For example, the average weekly prevalence of a severe health problem was three times higher and the average number of days when dancers felt unable to dance was more than twice as high in company A than in company E.

Further, there seems to be a systematic pattern indicating an association of health problems and workload. In company E, the dancers rated the severity of all investigated variables on average lowest: they had the lowest seasonal and weekly prevalence of severe musculoskeletal pain and health problems, the lowest average number of days on which the dancer felt unable to dance and fewer dancers rated their physical and mental workload higher than “ideal” than in the other four companies. On the contrary, company A had the highest average weekly prevalence of health problems and of impaired ability to dance, as well as the highest average number of days when the dancer felt unable to dance. Company B had the highest average weekly prevalence of severe musculoskeletal pain, and the severity ratings of poor recovery after dancing, being stressed/overloaded and difficulty concentrating, and the second highest average number of days on which the dancer felt affected in their ability to dance or unable to dance. In these two companies (A & B) a higher percentage of dancers rated their physical and mental workload higher than ideal” than in the other companies.

## Discussion

This study used weekly self-reports to determine the prevalence of health problems in 98 professional ballet and contemporary dancers during the season and is the first that compared health problems between sexes and between five companies. In total 3123 health reports covering 21,861 dancer-days were received (response rate 74.1%). Dancers reported a health problem in 63% of the weekly reports, thus, more often than in every second week. Most health problems were injuries (76.7%), while illnesses (13.2%) and mental health problems (10.1%) were less frequent. Most variables were similar in female and male dancers, however the average weekly prevalence of severe health problems was about twice as high in female than in male dancers. Clinically significant differences between companies were observed for almost all investigated variables and indicated an association between health problems and workload.

### Health Problems and Impaired Ability to Dance

All dancers reported some degree of musculoskeletal pain and health problems and 90.0% felt that their ability to dance at full potential was impaired at least once during the season. This is similar to the prevalence of all health problems of dance students [14], of injuries of ballet dancers reported in a systematic review [1], of medical attention injuries of dancers at the Royal Opera House in London [5], and of all musculoskeletal injuries of professional dancers from three German companies [18]. The season prevalence of time-loss health problems in the present study (74.5%) was slightly higher than the season prevalence of time-loss injuries at the Royal Opera House [5], most probably since the present study also included illnesses and mental health problems. It is also possible that the different data collection methods (reports by in-house physiotherapists [5] versus self-reports) contributed to the observed difference. In agreement with the latter study [18], dancers reported a health problem more often than in every second week and felt affected in their ability to dance at full potential due to health problems in about half of the weeks. However, the percentage of days on which dancers felt impaired to dance at their full potential (23%) or completely unable to dance (6%) was lower in the present study than in the study conducted in the 2021/22 season in three German companies (29% days with impaired dancing ability, 10% days unable to dance) [18]. This might be due to a decrease in illnesses, especially COVID-19 infections and/or differences between companies as discussed below. The severity of all health problems and of impaired ability to dance at full potential were highly correlated, indicating the effect of health problems on the performance of dancers.

### Differences between Female and Male Dancers

In the present study, the season prevalence of musculoskeletal pain and of health problems was similar in female and male dancers as reported previously for injuries [5, 18]. Reports on sex differences in the incidence or incidence proportion of injuries are inconsistent in the literature [1, 5, 18, 22, 23]. Female dancers in the present study had a higher average weekly prevalence of severe health problems, and saw a medical professional in more weeks when they had a health problem than their male counterparts. Thus, female dancers seem to have more severe health problems but not injuries than male dancers. Future studies should analyse the prevalence and severity of injuries, illnesses and mental health problems to provide a more comprehensive picture of the full burden of health problems in professional dancers.

### Differences between Companies

The five companies included in the present study differed in almost all health-related variables. This further supports the results of a cross-sectional study [3] that showed considerable differences in injury prevalence between companies and has important implications for the interpretation of epidemiological studies as well as for prevention of health problems. For example, the season prevalence of severe musculoskeletal pain varied between 65.5% and 92.3%, and the average weekly prevalence of severe musculoskeletal pain between 5.5% and 17.7%. Thus, prevalence and characteristics of health problems found in one company should not be generalised to other companies. Possible reasons for the differences between companies (e.g. workload, size, training style, or repertoire) should be regarded in the prevention of health problems.

Several studies have investigated characteristics of the individual dancers as risk factors for health problems [24–29] but the influence of the company (e.g. size, work organization) has not been investigated previously, as most studies only included dancers from one company or analysed differences in dance style [30]. In the present study most dancers danced ballet and just one company danced contemporary. Although this company performs a different dance style and repertoire than the other four companies, it was not notable in terms of health problems or workload.

The results of the present study also indicated an association between subjective workload and health problems, i.e. more health problems in companies with higher (physical and mental) workload. Such a relationship has been described by dancers [18, 19, 31] and has also been found in quantitative studies [29, 32]. Byhring & Bo [31] reported that dancers believed that the risk of injury was related to training, organizational and environmental factors, and Bolling et al. [19] that dancers perceived the imbalance between workload and their capacity to manage the load as the main cause of injury. Further, dancers from three German companies stated that “too much workload” and “tiredness / exhaustion” and “stress / overload / insufficient regeneration” were the main reasons or causes of injury [18]. A significant association between the number of injuries and total dance exposure per month were also found in a prospective study on 66 elite full-time pre-professional dance students in New Zealand [32], while other studies have inconsistent results on the relationship between health problems and training load [33–35]. However, a large study on ballet dancers at the Royal Opera House in London found positive relationships of week-to-week changes in exposure with the risk of overuse time-loss and medical attention injuries as well as a negative relationship of accumulated exposure over seven days with the risk of overuse medical attention injuries [29].

### Strength and Limitations

Since participation in the project was voluntary, just 44.7% of the dancers employed at the five companies could be included in the study. However, 81.7% of the dancers who agreed to participate in the study answered at least 25% of the weekly health reports during the season, and the response rate to the weekly health reports was 74.1%. Thus, the results are likely to present a true picture on the health problems of the participating dancers and companies. Furthermore, the prevalence of health problems in the present study was similar to the prevalence of health problems and/or injuries reported in previous studies [5, 14, 18]. Although confidentiality was assured, it is possible that some dancers had not reported all their medical problems because of fear of potential consequences [13, 36], but this also applies to reports from in-house medical staff when dancers want to cover their medical problems [3]. However, most of the German opera houses and states theatres don´t have in-house medical staff, and thus, using self-reports was the only way to collect data on non-time-loss medical problems [18]. In the present study exposure data were not collected, and therefore, the incidence of health problems could not be calculated. However, the present study analysed the average weekly prevalence, which better reflects the burden of health problems.

## Conclusion

Health problems are frequent in professional dancers and affect their ability to dance. The prevalence of health problems varied between companies in a clinically significant way and seemed to be influenced by the physical and mental workload of the dancers.

Future studies should analyse the impact of physical and mental workload on health problems and develop related prevention strategies. The working conditions, structure and the medical care network of the company need to be considered when developing preventive measures to reduce the burden of health problems in professional dancers.

## Electronic Supplementary Material

Below is the link to the electronic supplementary material.

Supplementary Material 1

## Data Availability

Due to confidentiality reasons, no data can be shared.

## Abbreviations

NRS: Numeric Rating Scale
PAHM: Performing artist and Athlete Health Monitor
SD: Standard Deviation
SPSS: Statistical Package für Social Sciences
