# Supplementary material for: Self-reported Health Problems of Professional Dancers from Five German Opera Houses or State Theatres: A Prospective Study with Weekly Follow-ups during One Season
Source: Sports Med Open. 2024 Nov 9;10:121. doi: 10.1186/s40798-024-00782-w (PMC11550303; doi:10.1186/s40798-024-00782-w)
Supplement: Supplementary file 1 — Supplementary Material 1 [file 40798_2024_782_MOESM1_ESM.docx]

**Appendix:**

**Dancer´s weekly self-report of health complaints**

Note: This is not the entire questionnaire presented to the dancers but all questions analysed in the related publication.

Comments in red are logic for the software engineer, and not presented to the dancers.

**Dancer´s identification: Date of report:**

1. How do you rate your physical and mental / psychological **workload** in the **past 7 days**?

|  | much much  too low ideal too high |
| --- | --- |
| **physical** workload | -5        -4        -3        -2        -1        0        +1        +2        +3        +4        +5 |
| **mental** workload | -5        -4        -3        -2        -1        0        +1        +2        +3        +4        +5 |

◻ no dance activities in the **past 7 days**

*2.* Overall, **how severe** have your **musculo-skeletal pain or complaints** been in the last 7 days?

*Please rate on the scale from “no musculo-skeletal complaints” (0) to “worst imaginable” (10).*

no musculo-skeletal
complaints worst imaginable

0        1        2        3        4        5        6        7        8        9        10

*In the following the term “****health problems****” summarises all kinds of pain, complaints, injuries, illnesses and mental health issues.*

*3.* **How severe** have **all your health problems** (*all kinds of pain, complaints, injuries, illnesses and mental health problems*) in total been **in the past 7 days**?

*Please rate on the scale from “no health problem” (0) to “worst imaginable” (10).*

no health problem worst imaginable

0        1        2        3        4        5        6        7        8        9        10

*(If the answer to question 3 is “0”, ask questions 4 and then go to “Thank you!”-page)*

4. How much have all your health problems in total **affected your ability to dance** **at your full potential** in the **past 7 days**?

*Please rate on the scale from “0= not affected at all, I could dance at full dance potential” to “10= I was complete unable to dance (train, rehearse or perform) due to health problems”.*

dance at

full potential unable to dance

0        1        2        3        4        5        6        7        8        9        10

5. Have you seen a **physician, physiotherapist, psychologist or another qualified medical practitioner** because of your health problem(s) in the past 7 days?

*(multiple answers possible)*

◻ no

◻ yes, physician

◻ yes, physiotherapist

◻ yes, other please specify _____________________________ (*free text)*

6. On **how many** of the past 7 **days** have all your **health problems** in total **affected your ability to dance** at your full potential?

*Please consider all 7 days, even if no training, rehearsal or performance was scheduled.*

____ days of the past 7 days *(A number between 0 and 7 should be entered here.)*

7. On **how many** of the past 7 **days** have you been **completely** **unable to train, rehearse or perform** due to your health problem(s)?

*Please consider all 7 days, even if no training, rehearsal or performance was scheduled.*

____ days of the past 7 days *(A number between 0 and 7 should be entered here.)*

8. Please classify your **most severe** health problem:

◻ **musculo-skeletal pain / complaints** or **injury** (e.g. sore muscles, ankle sprain, concussion)

◻ **illness** (e.g. influenza, diarrhoea) or **physical symptoms** (e.g. headache, menstrual pain)

◻ **mental health** issue (e.g. performance anxiety, depression)

9. Would you like to **add more information** about your health problem(s) or is there anything else we should know?

◻ no

◻ yes please specify ________________________________ (*free text)*

**Thank you for taking the time to fill in the questionnaire!**
